# Successful application of prime and pull strategy for a therapeutic HSV vaccine

**DOI:** 10.1038/s41541-019-0129-1

**Published:** 2019-08-01

**Authors:** David I. Bernstein, Rhonda D. Cardin, Fernando J. Bravo, Sita Awasthi, Peiwen Lu, Derek A. Pullum, David A. Dixon, Akiko Iwasaki, Harvey M. Friedman

**Affiliations:** 1 Department of Pediatrics, University of Cincinnati College of Medicine, Cincinnati, OH USA; 2 School of Veterinary Medicine, Louisiana State University, Baton Rouge, LA USA; 3 Infectious Disease Division, Department of Medicine, Perelman School of Medicine, University of Pennsylvania, Philadelphia, PA 19104-6073 USA; 4 Department of Immunobiology, Yale University, New Haven, CT USA; 5 Howard Hughes Medical Institute, Chevy Chase, MD USA

**Keywords:** Preclinical research, Experimental models of disease

## Abstract

One promising approach for a herpes simplex virus vaccine uses a vaccine to prime and a chemoattractant to pull immune cells into the genital tract. We evaluated subunit vaccines (prime) and imiquimod (pull) in the guinea pig (gp) model of recurrent Herpes simplex virus type-2 (HSV-2). Following vaginal HSV-2 infection, gps were vaccinated with various combination of glycoproteins and adjuvant with or without subcutaneous or local applications of imiquimod after infection. Animals were examined daily for recurrent lesions and vaginal swabs collected for recurrent shedding. Although both the vaccines alone and imiquimod alone reduced recurrent HSV disease, the combination of local imiquimod and vaccine (Prime and Pull) was the most effective. In the first study, immunization with the trivalent vaccine alone or imiquimod alone decreased recurrent disease. However, the largest decrease was with the combination of vaccine and local imiquimod (*P* < 0.001 vs. placebo or vaccine alone). No effect on recurrent shedding was observed. In the second study, recurrent disease scores were similar in the PBS control group and the trivalent-immunized group treated with subcutaneous imiquimod however, significant reductions with glycoprotein vaccines and local imiquimod (*p* < 0.01 vs. placebo) were noted. The number of qPCR-positive recurrent swabs, ranged from 5 to 11% in the vaccinated+local imiquimod groups compared 29% in the PBS control group (*P* < 0.05). No recurrent swab samples from vaccinated groups were culture positive. We conclude that the strategy of prime (subunit HSV vaccine) and topical pull (intravaginal/topical imiquimod) decreased recurrent HSV more effectively than vaccine alone.

## Herpes simplex virus: prime and pull vaccination

“Prime and pull” vaccination strategies involve an initial vaccination followed by the local application of a stimulant such as chemokines to recruit immune cells to infection target areas such as the mucosa. David Bernstein and colleagues investigate the efficacy of a prime and pull approach in a guinea pig model of recurrent genital Herpes simplex virus 2 (HSV-2) infection. Animals were vaccinated with HSV-2 glycoproteins in adjuvant with or without subcutaneous or topical (vaginal) exposure to the immune stimulant imiquimod to act as the “pull”. Animals with topical imiquimod show superior control of HSV-2 infection and improved recruitment of CD8^+^ T cells to the vaginal epithelium. Topical application of imiquimod demonstrates better control than subcutaneous imiquimod suggesting that “pulling” might be a useful approach in HSV vaccination.

## Introduction

Herpes simplex virus type 2 (HSV-2) is estimated to infect 417 million people globally, with 19.2 million new infections annually.^1^ Almost all infected individuals develop recurrences, either recurrent lesions or even more often recurrent shedding in the absence of lesions.^2,3^ Asymptomatic shedding occurs frequently, > 10% of days and is believed to be the major cause of transmission.^2–4^ Further, neonatal HSV infections produce a serious disease with high rates of morbidity and even mortality despite current antiviral therapies.^5,6^ Genital HSV infections are also recognized to increase the acquisition of HIV and result in increased shedding of HIV.^7^

Available antiviral therapies are somewhat effective in the treatment of recurrent HSV disease; shortening lesion duration and decreasing viral shedding but are only effective during the time of drug administration.^8,9^ Therefore, there has been great interest in developing therapeutic vaccines designed to decrease lesion rates as well as shedding rates.^10^ The evaluation of immunotherapy for recurrent herpesvirus infection dates back to at least the 1930s. However, the therapeutic potential of immunotherapy was not demonstrated conclusively until 1988, when Stanberry et al.^11^ were able to show, using the guinea pig model of genital herpes, that a vaccine could indeed reduce the number of genital herpes recurrences. This preclinical model was later extended to show the effects of immunization on recurrent shedding.^12^ Most recently, this preclinical work was supported by several clinical trials^13,14^ of a bivalent HSV-2 vaccine provided with a potent adjuvant. In these trials, vaccination reduced recurrent lesions or recurrent shedding rates by ~ 50%.

We sought to improve upon this rate by employing a prime and pull strategy.^15^ The prime and pull strategy relies on two steps: (1) conventional parenteral vaccination to elicit systemic T-cell responses (prime), followed by 2) recruitment of activated T cells via topical administration of a T cell attractant (pull), where such T cells establish long-term protective immunity. The original description applied to prophylactic HSV immunization and showed the primary CD8 T-cell responses were of similar magnitudes in the spleen, whereas the frequency and number CD8 T cells in the vagina were significantly higher in vaccinated mice treated with a vaccine and the chemokine pull as compared with the control immunized mice without the “pull”. Furthermore, the action of the chemokine pull was restricted to the genital mucosa, as CD8 T-cell recruitment to the vagina-draining lymph nodes was limited. Importantly, the prime and pull strategy conferred near complete protection against primary challenge with genital HSV-2 infection compared with prime alone.^15^ In this manuscript we extend the application to therapeutic vaccines and show that the frequency of recurrent disease and recurrent vaginal shedding was reduced most effectively by the combination of prime (glycoprotein vaccine) and pull (vaginal imiquimod).

## Results

### Study 1

**Fig. 1:**
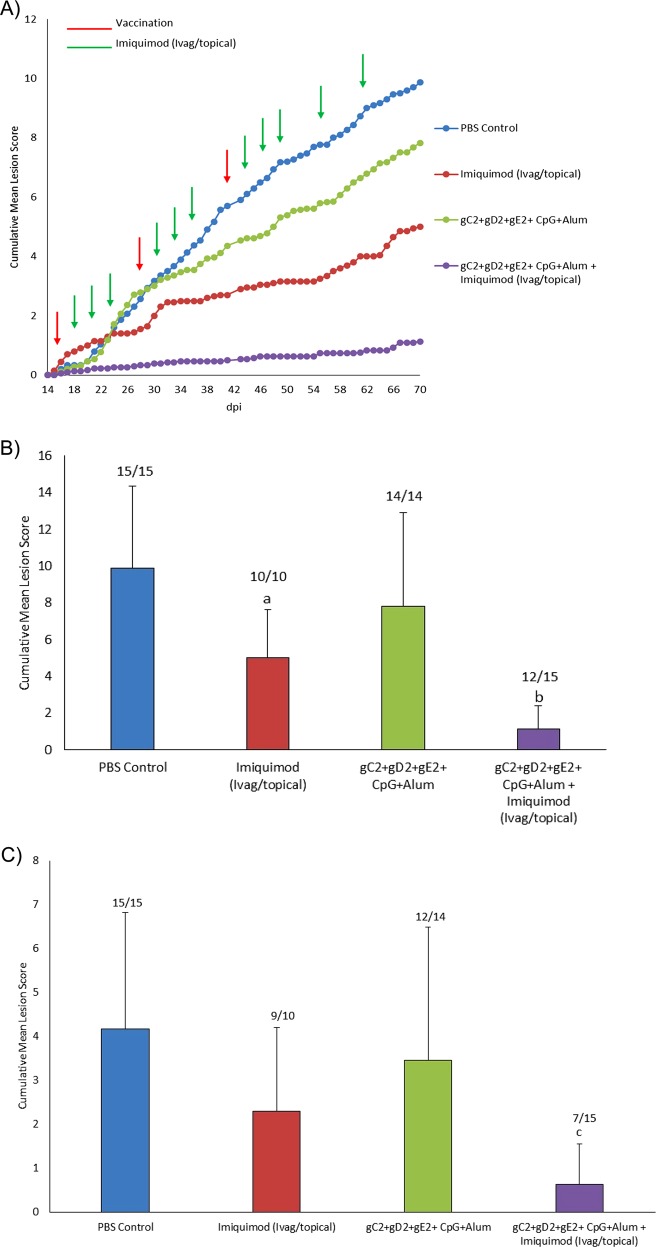
Recurrent Lesion Scores. In study 1, HSV-2 infected animals were either vaccinated with trivalent vaccine, treated Ivag/topical with imiquimod, both or neither as described in the methods. Animals were then followed daily for recurrent lesions from day 15–70. **a** shows the cumulative recurrent lesion scores over the time period of day 15–70. **b** shows the total recurrent lesion score from the day after the first immunization (day 15) until day 70. **c** shows total recurrent lesion score from the day after the third immunization (day 43) until day 70. The numbers above the bars are the number of animals with recurrent lesion/total number of animals ^a^*p* < 0.05 vs PBS; ^b^*p* < 0.001 vs vaccine alone group ^c^*P* < 0.001 vs. PBS and *P* < 0.01 vs the vaccine alone group comparing mean lesion scores

In study one (Fig. 1), immunization with the trivalent vaccine plus adjuvant decreased recurrent disease scores from 9.9 ± 4.5 in the placebo group to 7.8 ± 5.1 but the decrease was not significant (Fig. 1b). Treatment with imiquimod (Ivag/topical) alone also decreased recurrences (5.0 ± 2.6, *P* < 0.02 vs. placebo) but the largest decrease was with the combination of vaccine and imiquimod (Ivag/topical). This group had significantly reduced recurrent lesion scores (1.1 ± 1.3) compared with the placebo group as well as to the vaccine alone group (*P* < 0.001 to each) (Fig. 1b). No evidence of local irritation or inflammation was observed during the treatment with imiquimod. Similarly, after the three vaccinations recurrent lesion scores were significantly lower in the vaccine and imiquimod (Ivag/topical) group compared with the placebo (*P* < 0.001) or vaccine alone group (*p* < 0.01) (Fig. 1c).

No significant differences were found between groups in the number of animals that developed recurrent lesions (*P* ≥ 0.20, Fig. 1b) when the entire period of observation was evaluated. However, the number of animals with recurrences was significantly lower (*P* < 0.02) in the gC2/gD2/gE2+imiquimod group (Ivag/topical) in both the period after the second dose (9/15, 60%) and after the third dose (7/15, 47%, Fig. 1c) when compared to the PBS group (15/15, 100% for both periods).

**Fig. 2:**
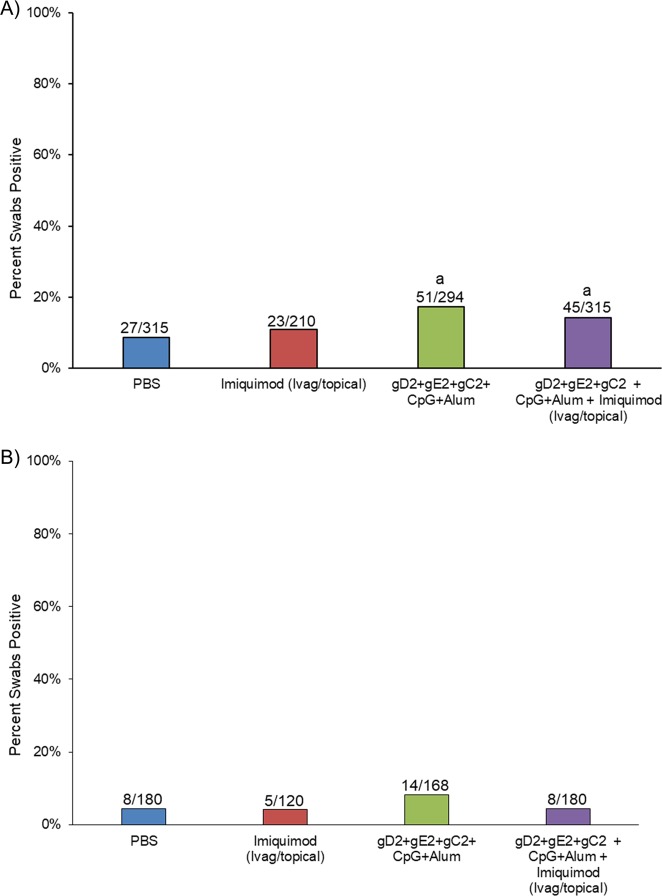
Percent (number of recurrent swabs with detectable HSV DNA). In study 1, HSV-2 infected animals were either vaccinated with trivalent vaccine, treated Ivag/topical with imiquimod, both or neither as described in the methods. Vaginal swabs were then collected three times per week from day 21–70. Swabs were evaluated by qPCR for the presence of HSV DNA and the number of swabs that were positive/total number of swabs for each group is presented above the bars and plotted as the per cent positive. **a** shows the total number of positive swabs from the day after the first immunization (day 15) until day 70. **b** shows the number of positive swabs the day after the third immunization (day 43) until day 70. ^a^*p* < 0.05 vs placebo

An effect on the number of days with recurrent vaginal shedding of HSV-2 DNA was not seen in this experiment (Fig. 2). In fact, the number of days with recurrent shedding was significantly increased in the groups receiving the gC2/gD2/gE2 vaccine or the gC2/gD2/gE2 vaccine+imiquimod compared with placebo over the entire period (*P* < 0.05) Fig. 2a. The comparison after the third immunizations shows that shedding was more similar and not significantly different comparing vaccinated groups to placebo (Fig. 2b).

**Fig. 3:**
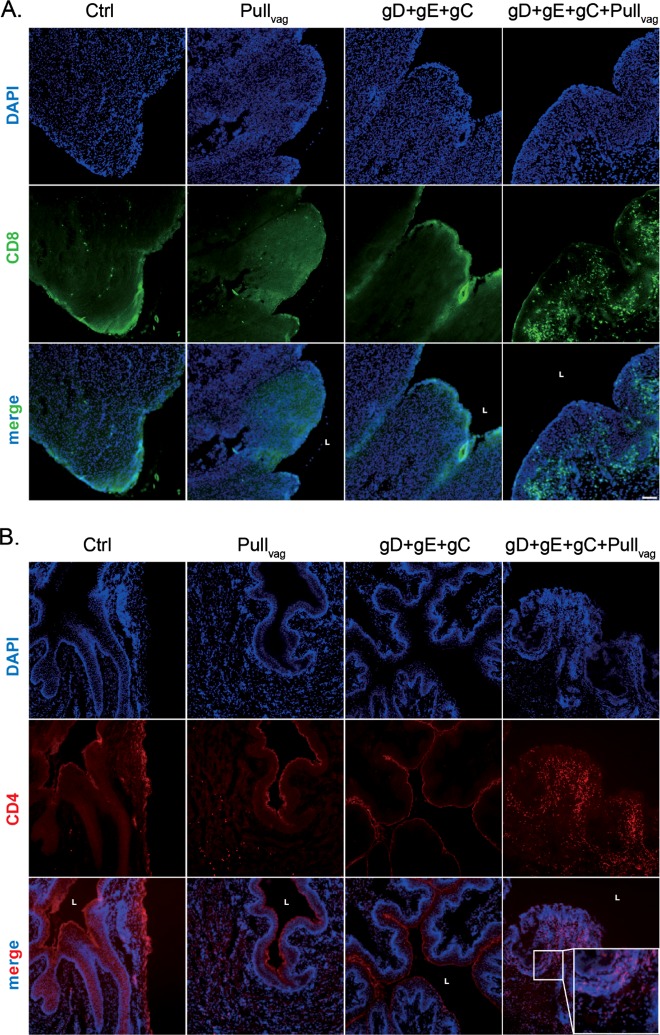
CD4 and CD8 T-cell localization in the vagina of guinea pigs that received trivalent vaccine Prime with or without Ivag/topical imiquimod Pull. Vaginas from four groups were collected at the termination of the study (day 70) and frozen in OCT. Frozen sections were stained with antibody to CD8 and CD4 and analyzed by fluorescence microscopy using the ×10 objective lens. Scale represents 100 μm. **a** CD8 staining **b** CD4 staining. The L notation marks the lumen of the vaginal cavity

Analysis of the frozen vaginal sections showed the effect of the prime and pull strategy on the number of CD4 but especially CD8 T cells in the vaginal tissues as compared with either the placebo, trivalent vaccine (gD+gE+gC) alone or the pull (imiquimod) alone). Thus, although some CD8 T cells were detected in all groups, the animals vaccinated with gC2/gD2/gE2+imiquimod Ivag/topical had an increased number of CD8 T cells, particularly within the epithelial layer (Fig. 3a). We also observed an increase in the number of CD4 T cells in the submucosa within the group receiving gC2/gD2/gE2+imiquimod Ivag/topical treatment (Fig. 3b).

### Study 2

**Fig. 4:**
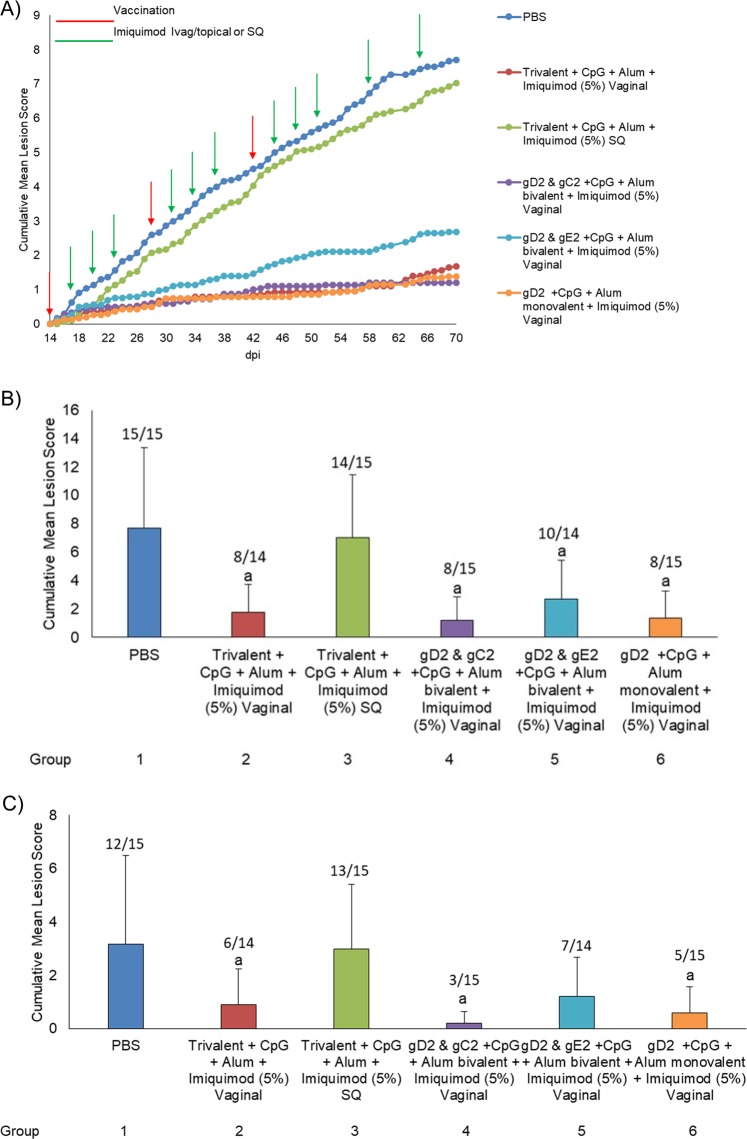
Recurrent lesion scores for Study 2. In study 2, HSV-2 infected animals were either unvaccinated (PBS), vaccinated with trivalent vaccine and treated SQ with imiquimod, vaccinated with trivalent vaccine and treated Ivag/topical with imiquimod, vaccinated with bivalent or monovalent vaccine and treated Ivag/topical with imiquimod. Animals were then followed daily for recurrent lesions from day 15–70. **a** shows the cumulative recurrent lesion scores over the time period of day 15–70. **b** shows the total recurrent lesion score from the day after the first immunization (day 15) until day 70. **c** shows total recurrent lesion score from the day after the third immunization (day 43) until day 70. The number above the bars shows the number of animals with recurrent lesions/ total number of animals. ^a^*p* ≤ 0.05 vs group 1 and 3

In the second experiment we sought to determine if the trivalent vaccine was more effective than the monovalent gD2 or bivalent vaccines and whether parenteral (SQ) imiquimod would be as effective as the local (Ivag/topical) administration, thus providing evidence as to whether the effects were due to the adjuvant activity of imiquimod or the pull of effector cells into the genital tract. All vaccinated groups that were treated Ivag/topically with imiquimod showed a significant decrease (*p* < 0.01) in recurrent disease scores (ranging from 1.2–2.7 ± 1.7–2.7) when compared with the control PBS group (7.7 ± 5.6) or the trivalent vaccine group treated with SQ imiquimod (7.0 ± 4.4) (Fig. 4). There were no significant differences (*p* > 0.50) compariing groups receiving Ivag/topical imiquimod+trivalent (gC2+gD2+gE2), bivalent (either gD2+gC2, or gD2+gE2), or monovalent vaccine (gD2) (Fig. 4a, b). Similarly, recurrent lesion scores remained significantly lower (*p* < 0.05) in all the vaccinated groups that were treated Ivag/topically with imiquimod during the period after the third vaccination (Fig. 4c). No evidence of local irritation was observed during the treatment with imiquimod.

All of the vaccinated groups receiving Ivag/topical imiquimod also had a significant reduction in the percentage of animals with recurrent lesions (ranging from 53 to 67%) when compared with the control PBS group (15/15, 100%, *p* < 0.05) (Fig. 4b). The reduction after all three immunizations (days 43–70) is even greater (Fig. 4c). With the exception of the gD2 & gE2+CpG+Alum (group 5), all of the groups receiving vaginal imiquimod also had a significant reduction in the percentage of animals with recurrent lesions when compared with the trivalent vaccine groups receiving SQ imiquimod (group 3, *p* < 0.05). Similar to the lesion scores, there were no differences in comparisons made between the groups treated vaginally with imiquimod.

**Fig. 5:**
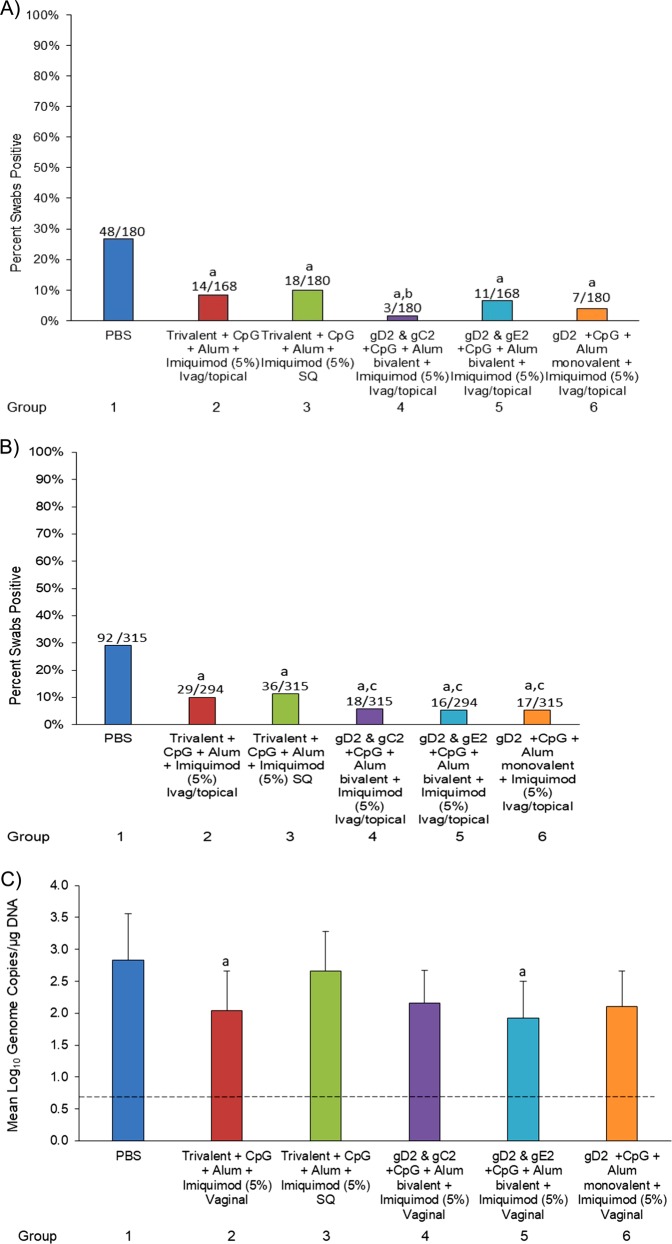
Recurrent vaginal shedding. In study 2, HSV-2 infected animals were either unvaccinated, vaccinated with trivalent vaccine and treated SQ with imiquimod, vaccinated with trivalent vaccine and treated Ivag/topical with imiquimod, vaccinated with bivalent or monovalent vaccine, and treated with Ivag/topical imiquimod as described in the methods. Vaginal swabs were then collected three times per week from day 21–70. Swabs were evaluated by qPCR for the presence of HSV DNA. **a** shows the number of swabs that were positive/total number of swabs for each group as presented above the bars and plotted as the per cent positive for the entire period of swabbing (d21–70). **b** shows this information for the period after the third immunization (day 43–70). **c** shows the quantity of HSV-2 DNA in the positive swabs from day 21–70 with the dashed line representing the limit of detection. ^a^*p* < 0.001 vs group 1; ^b^*p* < 0.05 vs group 2 ^c^*p* < 0.05 vs. group 3

Recurrent shedding was also significantly reduced. The number of positive swab samples was lower in all of the vaccinated groups, ranging from 5 to 11%, 16–36/294–315, *p* < 0.05). when compared with the PBS control group 29% (92/315) (Fig. 5). In addition, all groups receiving Ivag/topical imiquimod had reduced shedding rates (5–6%) compared with the group receiving SQ imiquimod (11%). Lastly, the shedding rate in the monovalent gD2 group+Ivag/topical imiquimod was similar to and in effect lower than the trivalent or bivalent groups+Ivag/topical imiquimod (Fig. 5a). Importantly, none of the vaccinated groups shed detectable (infectious) virus when evaluated by culture compared with the PBS group where virus was recovered from 1.2% of the swabs. Evaluating shedding after the third immunization provided similar results as all the vaccinated groups had siginficanty (*p* < 0.001) fewer positive swabs than the PBS group (Fig. 5b).

Further, the quantity of HSV-2 DNA in the swabs that were positive was lower in each of the Ivag/topical imiquimod groups (1.93–2.16 ± 0.51–0.62 mean Log10 genome copies/µg of DNA) compared with the PBS control group (2.83 ± 0.73 mean Log10 genome copies/µg of DNA) (Fig. 5c). Two of the groups treated with Ivag/topical imiquimod (Trivalent and gD2+gE2) also had a reduction in the quantity of HSV-2 DNA (2.04 ± 0.62 and 1.93 ± 0.58 mean Log10 genome copies/µg of DNA, respectively) when compared to the Trivalent group treated with SQ imiquimod (2.66 ± 0.62 mean Log10 genome copies/µg of DNA (NS) (Fig. 5c).

The number of animals with at least one positive swab sample in the study was also analyzed. The bivalent group gD2+gE2 and the monovalent gD2 group treated with Ivag/topical imiquimod had the most animals without a positive swab (8/14, 57% and 8/15, 53%, respectively) as compared to both the PBS control group (0/15, 0%) and the trivalent group treated with SQ imiquimod 0/15, *p* < 0.05).

**Fig. 6:**
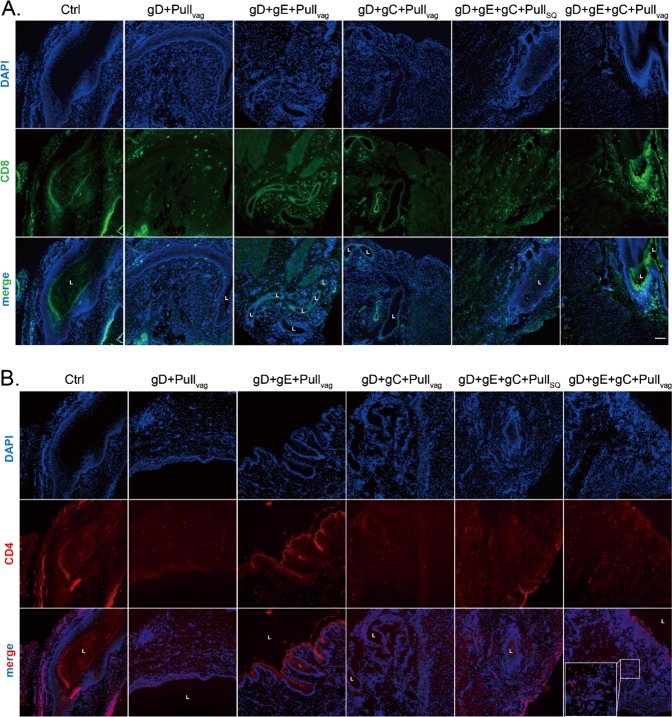
CD4 and CD8 T-cell localization in the vagina of guinea pigs that received monovalent (gD), bivalent (gD+gC or gD+gE), or trivalent (gD+gC+gE) vaccine with either SQ imiquimod or Ivag/topical imiquimod. Vaginas were collected at the termination of the study (day 70) and frozen in OCT. Frozen sections were stained with antibody to CD8 and CD4 and analyzed by fluorescence microscopy using the × 10 objective lens. Scale represents 100 μm. **a** CD8 staining **b** CD4 staining. The L notation marks the lumen of the vaginal cavity

Analysis of the frozen vaginal sections from experiment 2 again showed that groups administered vaccine+Ivagl/topical imiquimod had an increase in both CD4 but predominately CD8 T cells when compared with placebo or the group given vaccine and SQ imiquimod. The animals vaccinated with gC2/gD2/gE2+Ivag/topical imiquimod appear to have the most CD8 T cells (Fig. 6).

## Discussion

Reducing recurrent HSV disease using immunotherapy is a major goal for HSV vaccine developers. Recent efforts have used a bivalent HSV vaccine and adjuvant to achieve an ~ 50% reduction in recurrent HSV disease and recurrent shedding.^13,14^ Although this demonstrates the proof of concept for a therapeutic vaccine it falls somewhat short when compared to newer antiviral therapies, which can reduce recurrent disease and shedding by almost 90%.^16,17^ Nevertheless, the advantages of vaccine strategies, requiring infrequent administration less than yearly vs. drugs that must be given daily suggest that improving vaccine effectiveness is a worthwhile endeavor.

Previous work has shown that there is a population of CD8 T cells that can be found near nerve endings that control reactivated HSV.^18,19^ Further, in simulations the local CD8 T-lymphocyte density in the mucosa predicted the peak HSV DNA copy number and whether genital lesions or subclinical shedding occurred.^20^ Their model suggested that the rate of containment of infected cells by the peripheral mucosal immune system is the major driver of duration and severity of HSV-2 reactivation.

We therefore sought to utilize the prime pull strategy originally described for prophylactic HSV vaccines by Shin and Iwasaki^15^ to enhance the number of HSV specific T cells that would control reactivated HSV as it emerges from nerve endings to cause recurrent disease and recurrent virus shedding.

In the first study, the prime pull strategy significantly decreased recurrent HSV-2 disease compared with the prime (gD+gC+gE vaccine alone) or the pull (Ivag/topical imiquimod) alone but curiously, none of the treatments reduced recurrent vaginal shedding. In the second study, designed to compare the trivalent vaccine to a monovalent gD vaccine and systemic vs. Ivag/topical imiquimod, we found that any of the vaccines when given with Ivag/topical significantly reduced both recurrent disease and recurrent vaginal shedding. Importantly, none of the vaccinated animals shed detectable infectious virus (limit of detection five PFU/swab) during the period of observation for recurrences. We also found the monovalent vaccine was as effective as the trivalent or bivalent vaccines in reducing both recurrent disease and recurrent vaginal shedding and that Ivag/topical imiquimod was significantly more effective than systemic imiquimod. This later finding strongly suggests that imiquimod was acting more as a T cell attractant rather than as an adjuvant.^21^ This hypothesis is further supported by the immunofluorescent analysis that shows the increase in local CD8 T cells in the Prime and Pull treated guinea pigs.

It is not clear why the effects on HSV shedding were different between the two studies but it should be noted that the rate of shedding in the PBS group in the first study was < 10%, which is substantially less than we usually observe, and was less than in the second study (29%), making it difficult to detect an effect of vaccinations. Further, in the past shedding has been more difficult to affect than lesion development in this model for both antivirals^22^ and vaccines.^23,24^ It is also possible that the small sample size and the variability in recurrent disease and shedding could result in inconsistent results. In human trials hundreds of subjects are often enrolled while animal studies usually use 10–20 animals/group.

Although no local irritation was noted in the guinea pigs, imiquimod may not have an acceptable safety profile for topical application in humans. Topical imiquimod creates a localized immune response that can produce signs of local irritation including erythema, edema, induration, ulcerations, and crusting^25^ with ~10% of patients developing erosions and ulcerations at the application site.^26^ In addition, adverse reactions not only occur at the site of application, but also in the vicinity of application.^27^ For these reasons we are currently exploring other topical treatments, including lower doses of imiquimod, which would be less reactogenic but still enhance recruitment of T cells. Although the primary pull response was for CD8 T cells, we detected an increase in CD4 T cells that potentially poses an added risk of HIV acquisition. The use of CD8-specific pull, such as recombinant chemokines CXCL9 and CXCL10,^15^ or agents that selectively elicit these chemokines may circumvent such concerns in the future.

In summary, using glycoprotein vaccines to boost HSV immunity and intravaginal/topical imiquimod to pull these immune cells into the vaginal tract significantly reduced recurrent genital HSV lesions and the shedding of HSV-2 into the vaginal tract. These results reveal the effectiveness of the Prime and Pull vaccination strategy as it applies to therapeutic vaccines against genital herpes.

## Methods

### Vaccines

The baculovirus-expressed gC2 protein bac-gC2(426t) extends from amino acid 27 to amino acid 426, where amino acid 27 is the first amino acid after the signal peptide.^28,29^ The baculovirus-expressed gD2 protein bac-gD2(306t) extends from amino acid 26 to amino acid 331, where amino acid 26 is the first amino acid after the signal peptide.^29–31^ The methods used to construct bac-gD2(306t) resulted in an aspartic acid and a proline being added at the N terminus. The baculovirus-expressed gE2 protein bac-gE2(24-405t) extends from gE2 amino acid 24 to 405 where amino acid 24 is the first amino acid after the signal peptide.^32^

Vaccines were administered at 10 µg for each protein/animal intramuscularly in the quadriceps with a total volume of 50 μl per immunization.^29^

### Adjuvant

CpG oligonucleotide (5′-TCGTCGTTGTCGTTTTGTCGTT-3′) (Trilink Inc.) was administered using 100 μg CpG/guinea pig. Alum (Alhydrogel, Accurate Chemical and Scientific Corp.) was used at 240 µg/guinea pig.

### Imiquimod

Aldara (imiquimod)^21,33^ was purchased from Cincinnati Children’s Hospital pharmacy as a 5% cream and 0.1 ml applied intravaginally using a 1 ml syringe and 0.1 ml applied to the external genital skin (area not covered by hair) When administered systemically it was purchased from Sigma and prepared with 5% dimethylsulfoxide in PBS and administered subcutaneously as 0.5 mg/kg/dose in the upper shoulder (referred to as SQ).

### Study design

In the first evaluation, 60 female Harley guinea pigs (250–300 gms, Charles River Breeding Laboratories (Wilmington, MA)) were obtained and housed under AAALAC approved conditions at Cincinnati Children’s Hospital Medical Center. Animals were divided into the following four groups: (*N* = 15/group):

1. PBS control
2. Imiquimod Ivag/topical
3. gC2+gD2+gE+CpG+Alum (trivalent vaccine)
4. gC2+gD2+gE+CpG+Alum (trivalent vaccine)+imiquimod Ivag/topical

In the second experiment, 90 animals as described above were divided into the following six groups: (*N* = 15/group)

1. PBS control
2. gC2+gD2+gE+CpG+Alum (trivalent vaccine)+imiquimod, Ivag/topical
3. gC2+gD2+gE+CpG+Alum (trivalent vaccine)+imiquimod, SQ
4. gD2 and gC2+CpG+Alum (bivalent vaccine)+imiquimod, Ivag/topical
5. gD2 and gE2+CpG+Alum (bivalent vaccine)+imiquimod, Ivag/topical
6. gD2+CpG+Alum (monovalent vaccine)+imiquimod, Ivag/topical

After HSV-2 infection, immunizations with subunit proteins were performed three times at 2-week intervals.

Animals were infected by intravaginal administration of 1 × 10^6^ pfu, HSV-2 strain MS as previously described.^34^ After inoculation a vaginal swab was collected at 2 dpi to document infection. Only animals infected with HSV-2 were vaccinated. The vaccines contained 10 µg of each of antigen/animal, and were mixed with CpG (100 µg/animal) and 240 µg/animal of Alum. Vaccines were administered intramuscularly (IM) three times at 14, 28, and 42 days after infection. Imiquimod was applied topically, as described above, or administered SQ on days 3, 6, and 9 after each vaccinationand then administered once weekly beginning on day 16 after the last vaccine.

Animals were observed daily for evidence of local irritation and for recurrent lesions (days 15–70) and vaginal swabs were collected on Monday, Wednesday and Friday (days 21–70) for evaluation of virus shedding as previously described.^35,36^ Recurrences were assigned a value of 1.0 if a vesicle was observed and 0.5 if only a red lesion was observed. The mean lesion score was obtained by averaging the scores for each animal over the duration of observation. Cumulative lesion scores are the average of the scores over time

All animal investigations were approved by the Institutional Animal Care and Use Committee at CCHMC.

### Immunofluorescence analysis

At the end of the study (70 dpi), the animals were sacrificed. Vaginal tract was excised and then frozen in OCT compound (Sakura Finetek USA). OCT-embedded 8-μm cryostat sections mounted onto Superfrost Plus Slides (Electron Microscopy Sciences) were fixed with cold acetone before being blocked in 0.1 m Tris-HCl buffer with 1% fetal bovine serum. Phycoerythrin-conjugated anti-CD8 antibody (clone: CT6, Bio-Rad) or PE-conjugated anti CD4 antibody (clone: CT7, Bio-Rad) staining was followed by a secondary antibody, Rhodamine conjugated anti-R Phycoerythrin (Abcam). Slides were stained with 4′,6-diamidino-2-phenylindole (Sigma) and mounted with Prolong Gold Antifade reagent (Thermo fisher). All slides were analyzed by fluorescence microscopy (BX51; Olympus) with 10x lens. Imaging data were analyzed with Imaris 7.2 (Bitplane) and ImageJ 1.46r (NIH).

### PCR

Vaginal swabs were stored at −80 °C. DNA was isolated from 200 µl of vaginal swab media using QIAamp DNA Mini Kit (Qiagen #51306) according to manufacturer’s protocol. To determine the amount of HSV-2 shed, HSV-2 gG2 gene detection was performed by quantitative PCR. The gG2 primer and probe sequences were as followed:^35^ Forward: 5′-CGG/AGA/CAT/TCG/AGT/ACC/AGA/TC-3′; reverse: 5′- GCC/CAC/CTC/TAC/CCA/CAA/CA-3′; and probe: 5′-FAM-ACC/CAC/GTG/CAG/CTC/GCC/G -tamRA-3′.

Each PCR reaction contained 100 ng of DNA, 50 µm of each primer, 0.10 µm of FAM/tamRA fluorescent probe, and 10 µl of Taqman Gene Expression Master Mix (ABI) in a total volume of 20 µl reaction. PCR amplification of HSV-2 DNA was performed on a 7500 Fast Real-Time PCR system (ABI) using the following conditions: pre-incubation at 50 °C for 2 min and 95 °C for 10 min followed by 50 cycles consisting of a denaturation step at 95 °C for 15 s, annealing at 60 °C for 1 minute, and elongation at 72 °C for 10 s. A standard curve for each virus was generated with ten-fold serial dilutions of purified HSV-2 DNA (ATCC) containing 10^5^–10^0^ HSV-2 copies in 50 ng of uninfected guinea pig brain DNA. The limit of detection for HSV-2 was determined to be between 10^0^ and 10^1^ copies, with excellent linearity (*R* ≥ 0.98) over five logs of HSV genomic DNA content.

### Virus cultures from vaginal swabs

Frozen vaginal swabs were quickly thawed and swab media added to Vero cell monolayers in 24-well plates for 1 hour at 37 °C. Cells were overlaid with 0.5% methylcellulose in complete DMEM supplemented with 25 μg vancomycin.^37^ Plaques were counted 5 days later. The lower limit of detection was <5 PFU/ml.

### Statistical analysis

For comparison of the means, an analysis of variance was initially performed and if significant differences among all the groups were noted, a Tukey’s test to adjust for multiple comparison was used. Data are presented as means and standard deviations. Incidence data were compared by Fisher’s exact test. All comparisons are two-tailed with a *P* value of < 0.05 considered significant.

### Reporting summary

Further information on research design is available in the Nature Research Reporting Summary linked to this article.

## Supplementary information

Reporting Summary

## Data Availability

The data sets generated and/or analyzed during the current study are available from the corresponding author.

## Supplementary information

**Supplementary information** accompanies the paper on the *npj Vaccines* website (10.1038/s41541-019-0129-1).
